# Task Allocation and Sequence Planning for Human–Robot Collaborative Disassembly of End-of-Life Products Using the Bees Algorithm

**DOI:** 10.3390/biomimetics9110688

**Published:** 2024-11-11

**Authors:** Jun Huang, Sheng Yin, Muyao Tan, Quan Liu, Ruiya Li, Duc Pham

**Affiliations:** 1 School of Information Engineering, Wuhan University of Technology, Wuhan 430070, China; yinsen@whut.edu.cn (S.Y.); muyaotan@whut.edu.cn (M.T.); quanliu@whut.edu.cn (Q.L.); 2 School of Mechanical and Electronic Engineering, Wuhan University of Technology, Wuhan 430070, China; liruiya@whut.edu.cn; 3 Department of Mechanical Engineering, School of Engineering, University of Birmingham, Birmingham B15 2TT, UK; d.t.pham@bham.ac.uk

**Keywords:** end-of-life products, task allocation, human–robot collaborative disassembly, Bees Algorithm, disassembly sequence planning

## Abstract

Remanufacturing, which benefits the environment and saves resources, is attracting increasing attention. Disassembly is arguably the most critical step in the remanufacturing of end-of-life (EoL) products. Human–robot collaborative disassembly as a flexible semi-automated approach can increase productivity and relieve people of tedious, laborious, and sometimes hazardous jobs. Task allocation in human–robot collaborative disassembly involves methodically assigning disassembly tasks to human operators or robots. However, the schemes for task allocation in recent studies have not been sufficiently refined and the issue of component placement after disassembly has not been fully addressed in recent studies. This paper presents a method of task allocation and sequence planning for human–robot collaborative disassembly of EoL products. The adopted criteria for human–robot disassembly task allocation are introduced. The disassembly of each component includes dismantling and placing. The performance of a disassembly plan is evaluated according to the time, cost, and utility value. A discrete Bees Algorithm using genetic operators is employed to optimise the generated human–robot collaborative disassembly solutions. The proposed task allocation and sequence planning method is validated in two case studies involving an electric motor and a power battery from an EoL vehicle. The results demonstrate the feasibility of the proposed method for planning and optimising human–robot collaborative disassembly solutions.

## 1. Introduction

Remanufacturing can contribute to environmental protection, resource conservation, and socioeconomic well-being [1,2]. Remanufacturing is a significant component of a circular economy [3]. Disassembly is the first and probably most critical step in the remanufacturing and recycling of end-of-life (EoL) products [4,5]. Disassembly sequence planning involves obtaining the optimal disassembly sequence, improving efficiency by saving time and reducing costs [6,7]. Disassembly sequence planning is a challenging combinatorial optimisation problem [8]. Disassembly operations in remanufacturing are performed manually by human operators, automatically by robots, and semi-automatically by both humans and robots. Owing to the complexity of the task and variability in the condition of the product at the end of its service life, disassembly is usually carried out manually, which is labour-intensive, inefficient, and costly. Robotic disassembly and human–robot collaborative disassembly (HRCD) have been developed to improve efficiency and reduce costs [9]. However, although robotic disassembly can be more efficient and less costly, there are still situations where flexible, cognitive, and dexterous skills are needed and human operators’ assistance is required to complete the disassembly process. HRCD can combine both efficiency and flexibility [10,11].

Appropriate task allocation is critical for HRCD to generate and optimise disassembly sequences [12]. Task allocation is the assignment of different disassembly tasks to different disassembly resources, where disassembly resources in HRDC mainly include operators and robots or equipment. A utility value could be separately generated for humans and robots as a criterion to judge whether the task is suitable for operators or robots. In HRCD, the total disassembly time consists of the disassembly operation time, tool changing time, pose alteration time, and movement time [13]. Obstacle avoidance paths are employed in calculating the movement time. The obstacle avoidance paths between two components are difficult to consider at the beginning of the planning process for complex EoL products. The straight-line distance between them can be calculated to estimate travel time as a baseline for determining such paths. The optimal disassembly solution with obstacle avoidance paths can be obtained by setting the corresponding paths and comparing the combined cost required for different solutions.

As a population-based search algorithm, the Bees Algorithm (BA) mimics the food foraging behaviour of honey bees and has been widely utilised to solve optimisation problems [14]. Evaluation criteria combined with the Bees Algorithm were introduced to optimise robotic disassembly sequences [15]. The Bees Algorithm is based on the foraging behaviour of bees in nature, possessing powerful global search capabilities [16]. After each iteration, the scout bees, except for the elite scout bees, are reassigned to perform a global search. This will prevent the algorithm from being trapped in local optima. Its implementation is simpler than other complex optimisation algorithms. Complex parameter tuning is not required by the Bees Algorithm [17]; only the iteration number, the number of scout bees, and the number of employed bees need to be set. It exhibits low sensitivity to initial conditions and parameter settings, demonstrating high robustness and the ability to adapt to various uncertainties and changes [18,19]. The Bees Algorithm has great potential for the optimisation of HRCD sequence and task allocation.

This paper proposes the method of task allocation and sequence planning based on the Bees Algorithm for human–robot collaborative disassembly to improve efficiency. The article is organised as follows: Section 2 briefly reports related work. Section 3 introduces the assumptions, the different evaluation criteria for resources, and assigning strategies in task allocation. Section 4 describes the Improved Discrete Bees Algorithm for HRCD sequence planning, and the disassembly model is built to generate feasible disassembly sequences, and the concepts of forbidden direction, preferred direction, and optimisation objectives in disassembly sequence planning are introduced. Section 5 presents two case studies to validate the proposed method, involving an electric motor and a power battery from EoL vehicles. Finally, the conclusion is given in Section 6.

## 2. Related Work

### 2.1. Human–Robot Collaborative Disassembly

There is much reported research on disassembly sequence planning methods for EoL products [20,21]. The first step in the disassembly sequence planning process is to select a suitable disassembly mode, which can be classified as complete/partial disassembly or sequential/parallel disassembly [22]. After determining a disassembly mode based on the disassembly target, the next step is to generate and optimise disassembly sequences [23]. A disassembly sequence generation method using a constraint model based on a spatial interference matrix was presented [24,25]. To address the problem of infeasible disassembly sequences generated for EoL products with fasteners such as bolts, the spatial interference matrix was improved and a disassembly constraint model was established by Liu et al. [15].

Optimisation algorithms have been adopted to optimise disassembly sequences by maximising or minimising objective functions. To reduce disassembly time, a Particle Swarm Optimisation (PSO) encoding scheme was employed to define disassembly sequences in a matrix [26]. A disassembly sequence optimisation method based on the genetic algorithm (GA) was proposed by Maroua et al., which was a heuristic optimisation algorithm that simulates the processes of genetic inheritance, crossover, and mutation in biological evolution. The advantages of GA were computational speed and simplicity with minimal mathematical formulations [27,28]. Ant Colony Optimisation (ACO), a heuristic algorithm, was applied with a multi-layer representation method for disassembly sequence optimisation [29]. A self-adaptive simplified swarm optimisation (SASSO) algorithm combining the swarm optimisation algorithm with self-adaptive parameter control was investigated [30]. As a population-based search algorithm, the Bees Algorithm (BA) mimics the food foraging behaviour of honey bees and has been widely utilised to solve optimisation problems [31].

### 2.2. Disassembly Task Allocation Methods

Research relevant to the development of task allocation was reviewed and the general workflow was divided into six stages: task description and modelling, analysis and modelling of the task allocation process, algorithm selection for task allocation, decision-making of task allocation, simulation, and task execution [32]. A collaborative manufacturing task information model was developed based on the logical manufacturing unit and process [33]. A method concentrating on industry ergonomics and various task analyses is proposed [34]. The method was just based on 12 basic requirements, like the changes in workload. With the rise of the remanufacturing industry, research has also been conducted on the task allocation of disassembly sequence planning of EoL products.

In the field of disassembly sequence planning for HRCD, disassembly tasks should be assigned to different disassembly resources after generating the disassembly sequences. The allocation basis is often constituted by multiple facets. The components were classified into different disassembly tasks according to their characteristics, the disassembly tasks were divided into four categories, and suitable disassembly tasks were assigned to the operator and robot [35]. To target the right components based on remanufacturability factors, the PROMETHEE II method was employed to select the components based on cleanability, reparability, and economy [36]. Tasks were allocated according to their complexity and operators’ ergonomics by Lou et al. [12]. The disassembly tasks were classified according to the disassembly difficulty by Xu et al. [13].

In these references, some directly categorise the disassembly tasks based on component characteristics. In others, the maximum value of ranked attributes is used as the criterion for disassembly task allocation. Some provide disassembly task allocation standards between different levels of attributes but without specific descriptions. The studies do not cover detailed and comprehensive standards for disassembly task allocation. Compared to assembly, the criteria for task allocation in disassembly are not well-established. To fill these gaps, the present study has established detailed and comprehensive criteria for HRCD. Different criteria for evaluating human operators and robots are presented. Two case studies of EoL products are applied to substantiate the presented criteria and methods.

## 3. Task Allocation for Human–Robot Collaborative Disassembly

### 3.1. Objective and Work

**Figure 1:**
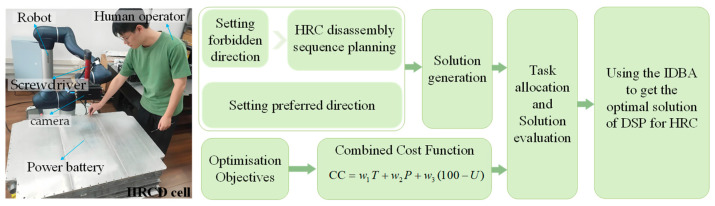
The workflow of the proposed method.

The paper’s objective was presented in the introduction section. The workflow of the proposed method of HRCD sequence and task allocation is shown in Figure 1. Before task allocation and solution evaluation, it is essential to develop a disassembly sequence and define the optimisation objectives. Following the establishment of forbidden directions, feasible disassembly plans are generated. Subsequently, by considering preferred directions, disassembly plans are tailored to emphasise specific directional biases. The optimisation objectives constitute a multi-objective optimisation problem, encompassing factors such as time, cost, and utility value. Upon completion of these preliminary steps, task allocation and solution evaluation are conducted. Finally, optimisation is performed by utilising the IDBA approach. The proposed method could be used for HRCD task allocation and disassembly sequence planning of EoL products with complex structures. As an example, a HRCD cell containing a human operator and a robot is also shown in Figure 1.

### 3.2. Assumptions

This section provides a concise and precise description of the experimental results, their interpretation, as well as the experimental conclusions that can be drawn. In the whole DSP process, some assumptions should be defined. There are various conditions and models in HRCD in the industry. The method developed in this research is based on the following assumptions.

1. It is assumed that all parts of the EoL products are disassembled completely, and non-destructively;
2. The disassembly resources in an HRCD cell consist of an operator and a robot. Robots can only be equipped with one device at a time. Each component can be disassembled by either the operator or the robot with different tools;
3. EOL products are sequentially disassembled. This means that the operator would wait for the robot to finish a task, or the robots would not perform the next disassembly task until the operator completes the last one. Taking the high human labour cost into consideration, the waiting time and operation time for human operators should be minimised, while the optimal strategy for HRCD task allocation should also consider the balance between time and cost;
4. When a component is disassembled, it should be immediately removed to prevent hindering the subsequent disassembly. The placement times for components by both the robot and human operator are simplified to a fixed duration;
5. Disassembly resources are represented, respectively, by digits 1–4. 1—indicates that both disassembly and placement of components are performed by robots; 2—indicates that both disassembly and placement of components are performed by operators; 3—indicates that disassembly is performed by robots and placement by operators; 4—indicates that disassembly is performed by operators and placement by robots;
6. The operator/robot can execute the next disassembly task after the previous disassembly task has been finished. The establishment of clear evaluation criteria is crucial for accurately assigning tasks within HRCD. With these criteria defined, we now turn to the foundational assumptions guiding our research approach.

### 3.3. Evaluation Criteria for HRCD

In disassembly, the continuity, safety, and flexibility of human operators’ and robots’ disassembly behaviours are not identical, necessitating different evaluation criteria for human operators and robots. Section 3.3 is divided into three parts: evaluation criteria for human operators, evaluation criteria for robots, and utility value calculation. The evaluation criteria for human operators include preliminary assessment, muscle fatigue, ergonomics, difficulty, flexibility, and repetition; those for robots include tool and capability preliminary assessment, assessment of disassembly difficulty, reachability assessment, and robot payload assessment.

#### 3.3.1. Evaluation Criteria for Human Operators

- Preliminary assessment

It is necessary to evaluate the environment and capacity for allocating tasks to humans to guarantee human operators’ safety [37]. The preliminary assessment involves the external situation, prospective danger during the disassembly process, or the possible operations exceeding human operators’ capacity. After a disassembly task passes the preliminary assessment, the following criterion is applied to calculate the utility value of the task, as shown in Table 1.

- Muscle fatigue

A new method concerning dynamic muscle fatigue is proposed in Ref. [38] to analyse the physical work and predict muscle fatigue. Several critical parameters and equations are shown in Table 2.

In the dynamic model, $F_{load}(t)$ is assumed to be constant. The relationship between duration and $f_{MVC}$ is shown in Equations (1) and (2) [38]:

(1) $$F_{cem}(t)=MVCe\int_{0}^{t}-k\frac{F_{load}(u)}{MVC}du=F_{load}(t)$$

(2) $$t=MET=-\frac{\ln\frac{F_{load}(u)}{MVC}}{k\frac{F_{load}(t)}{MVC}}=-\frac{\ln(f_{MVC})}{kf_{MVC}}$$

The model is compared with other existing general *MET* models [39], and two different correlation coefficients are introduced to calculate the relationship between the models’ outputs. The first is Pearson’s correlation coefficient r in Equation (3) [38], which indicates the linear relationship between the models’ outputs. The closer r is to 1, the more their outputs are linearly and positively related. The second is the intraclass correlation coefficient (*ICC*) in Equation (4) [38], indicating the similarity of the outputs of the two models. The closer ICC is to 1, the more similar their outputs are. In the context of model comparison, the ICC can be used to assess the consistency between the outputs or predictions of different models. This means that if the predictions of two models are highly consistent, the ICC value will approach 1, indicating that the two models’ predictions are similar.

(3) $$r=\frac{\sum_{n}\left(A_{n}-\bar{A})(B_{n}-\bar{B}\right)}{\sqrt{\sum_{n}{\left(A_{n}-\bar{A}\right)}^{2}{\left(B_{n}-\bar{B}\right)}^{2}}}$$

(4) $$ICC=\frac{{MS}_{betwenn}-{MS}_{within}}{{MS}_{between}+(q-1){MS}_{within}}$$

$A_{n},B_{n}$ are the *MET* of each method, and $\bar{A},\bar{B}$ are the average *MET* of each method. ${MS}_{betwenn}$ is the mean square between different MET values at different $f_{MVC}$ levels, and ${MS}_{within}$ is the mean square within MET values in different models at the same $f_{MVC}$ level. q is the number of models in the comparison. The fitness value related to muscle fatigue ($f_{H,mus}$) is shown in Equation (5) [38]:

(5) $$f_{H,mus}=\left\{\begin{array}{c} \frac{MAT}{MET},MAT<MET\\ 0,MAT>MET \end{array}\right.$$

- Ergonomics

The ergonomic factor is a complicated evaluation factor for human operators in disassembly tasks. More than ten segments are used in the ergonomic evaluation. Three main variables determine ergonomics, the type of posture, the duration of the time, and the force exerted externally. The software “ErgoToolkit v1” is introduced to evaluate ergonomic factors [40]. The implementation of the method consists of two main parts: a postural score sheet and a material handling score sheet.

Postural score sheet: This part contains 13 typical working body postures. Five different standards evaluate every posture: duration, basic scores (Table 3), twist scores, lateral scores, and reach scores (Table 4).

The material handling score sheet (Table 5): This criterion evaluates the material handling for every disassembly task. The critical parameters in the criterion are component dimensions, the magnitude of weight, and symmetry and operational difficulty of components.

The fitness value of the ergonomic factor is shown in Equation (6):

(6) $$f_{H,erg}=0.4\times f_{basic}+0.3\times f_{materaial}+0.1\times (f_{twist}+f_{lateral}+f_{reach})$$

- Difficulty and flexibility

This assessment consists of different evaluation factors, which show the complexity and requirements of a specific task for human operators. These factors are the requirements of tools for disassembly, accessibility of joints/grooves, and positioning [41], which is shown in Table 6.

Different types of tasks require various tools. The time spent on disassembly tasks might increase significantly as the tasks’ complexity rises. Therefore, the ideal situation for the operator is to complete a disassembly task without any tools. For complex disassembly tools, it takes more time for human operators to complete the task.

Accessibility of joints/grooves: This factor evaluates the availability of some typical features. The more inaccessible the features are, the more difficult it is for human operators to handle them. Positioning: This factor evaluates the level of accuracy required to position the tool. Due to the inherent nature of human beings, most human operators cannot perform tasks with very high accuracy requirements.

The fitness value of difficulty $f_{H,dif}$ is shown in Equation (7):

(7) $$f_{H,dif}=\frac{f_{requriement}+f_{Acess,d}+f_{Acess,l}+f_{Position}}{4}$$

- Repetition

This factor evaluates the repetition of tasks for human operators. The repetitiveness criterion is used to address the issue of operators repeating the same task over a long period. The criterion is quantitatively expressed as the maximum number of task repetitions performed by the same operator, as shown in Table 7. The fitness value ($f_{H,rep}$) could be obtained.

#### 3.3.2. Evaluation Criteria for Robots

- Tool and capability preliminary assessment

The tools installed on robots determine the robot’s capability in disassembly operations. The common tools and their corresponding capability when installed on robots are shown in Table 8. If the tool installed on the robot’s arm enables the robot to complete the disassembly operation, then the robot will pass the preliminary assessment. If not, the robot will change to the right tool.

- Difficulty assessment in disassembly tasks

The robots can deal with corresponding disassembly tasks with different robotic tools. The difficulty of a task and the corresponding fitness value are shown in Table 9. The difficulty fitness value: $f_{R,dif}$ is directly taken from Table 9.

- Reachability assessment

The robot’s arm is similar to that of human operators. The difference is that human operators can change their operation range much more easily than robots by changing their foot position. However, some collaborative robots (like massive load robots) are not flexible enough to change locations. The spheres are coloured according to their reachability index D to achieve this [42,43]. In most cases, when the disassembly parts are located at half the maximum reachability area, it is most convenient for robots to operate. If the robots cannot complete the disassembly task due to the shape of the components, this task will not be allocated to the robot. The reachability fitness value ($f_{H,reach}$) is from Table 10.

- Robot’s payload assessment

Although robots can complete those tasks with a weight less than the robot’s payload, in HRC disassembly tasks, robots should complete tasks whose weights are close to their payloads, leaving the lower-weight tasks to human operators. Therefore, the relationship between the weight of the task and the corresponding fitness value is shown in Equation (8) [44]. $R_{P}$ represents the maximum payload of the robot and $W_{P}$ represents the weight of disassembly components [44].

(8) $$f_{R,reach}=\left\{\begin{array}{c} 0,R_{p}<W_{P}\\ 1-\frac{R_{P}-W_{P}}{R_{P}},0.5<\frac{W_{P}}{R_{P}}<1\\ 0.5,R_{p}>W_{P} \end{array}\right.$$

#### 3.3.3. Utility Value Calculation

Different fitness values from Section 3.3.1 and Section 3.3.2 would be given different weights to calculate the final utility value. This value reflects whether a disassembly task is suitable for people or robots. Human operators and robots would not be allocated a disassembly task with a utility value lower than 0.5. The utility value formula for human operators and robots are presented in Equations (9) and (10).

(9) $$U_{h}=0.1\times f_{H,mus}+0.4\times f_{H,erg}+0.3\times f_{H,dif}+0.2\times f_{H,rep}$$

(10) $$U_{r}=0.33\times f_{R,dif}+0.33\times f_{R,reach}+0.33\times f_{R,pay}$$

### 3.4. Allocation Strategies

According to the disassembly model and criteria proposed in the article, the following five allocation strategies can be used.

(1) Randomised strategy: randomly allocating tasks with equal probability to the operator or robot.
(2) Equilibrium assignment strategy: if the former disassembly task is assigned to the operator/robot, this disassembly task is assigned to the robot/operator.
(3) Preference strategy based on utility value: prioritise allocating tasks to disassembly resources with higher utility values.
(4) Preference strategy based on payment value: prioritise allocating tasks to disassembly resources with higher payment values (the calculation method for Payment values will be introduced in Section 4.4.2).
(5) Preference strategy based on time value: prioritise allocating tasks to disassembly resources with higher time values (the time value for each disassembly task will be presented in Section 5.2 and Section 5.3).

These five allocation strategies are employed in the majority of studies, with allocation strategy one being used the most. The selection of allocation strategies should be random to ensure the consideration of all possibilities. Compared to strategy one, strategy two does not reflect the randomness of task allocation. Strategies three, four, and five can adjust the calculation coefficients of the final combined cost to achieve the same effect.

## 4. Sequence Planning for Human–Robot Collaborative Disassembly

### 4.1. Improved Discrete Bees Algorithm

**Figure 2:**
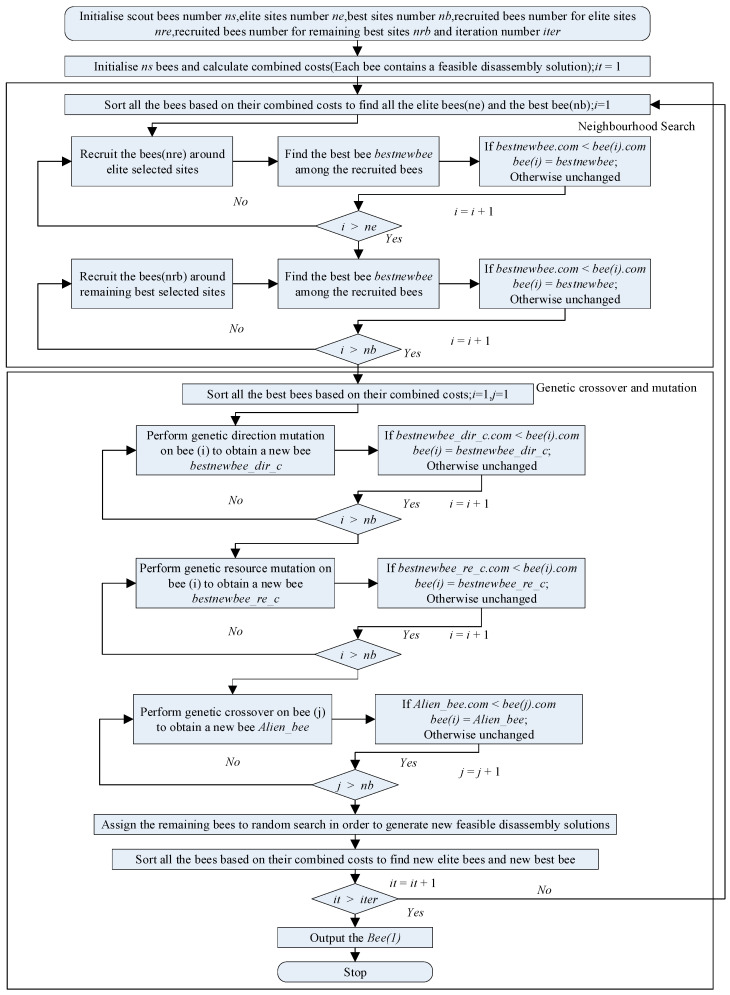
The workflow of IDBA.

The Improved Discrete Bees Algorithm (IDBA) primarily comprises initialisation, neighbourhood search, and genetic operations, as shown in Figure 2. Initially, the parameters of the Bees Algorithm need to be set. The parameter ns represents the initial number of scout bees, iter represents the number of iterations, mt represents the number of scout bees, ne represents the number of elite sites, nb represents the number of best sites, nre represents the number of employed bees for each elite site, and nrb represents the number of employed bees for each best site.

IDBA was developed based on the Enhanced Discrete Bees Algorithm (EDBA) [15]. IDBA combines EDBA with the priority preservation crossover operator, resulting in the introduction of two additional parameters that need to be initially set compared to the traditional Bees Algorithm. Mut represents the probability of the mutation operation occurring, and Cro represents the probability of the crossover operation occurring.

#### 4.1.1. Neighbourhood Search

**Figure 3:**
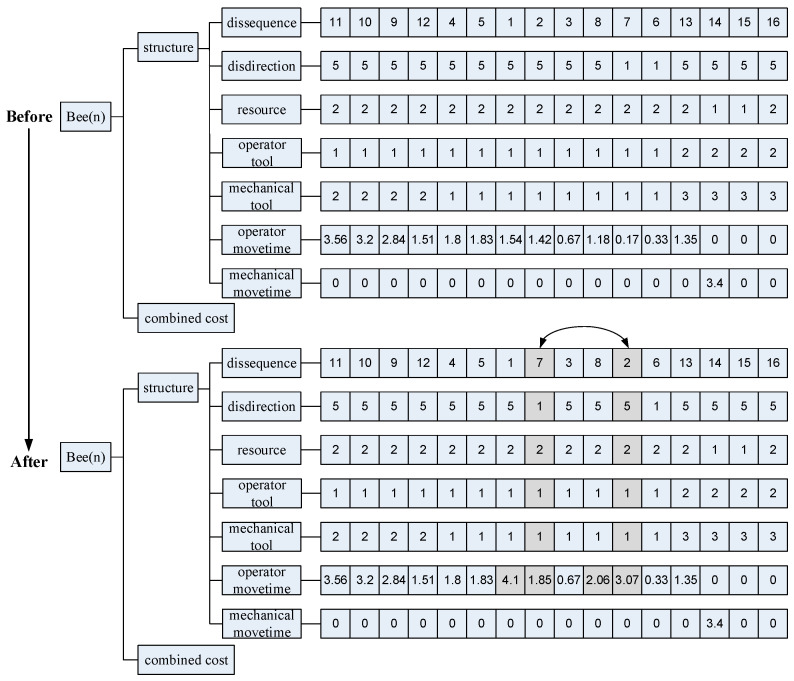
Swap operator in disassembly solution.

Neighbourhood search is a strategy in combinatorial optimisation aimed at finding the optimal solution within a defined solution space. Disassembly sequence planning presents a similar challenge in combinatorial optimisation to problems like the classical travelling salesman problem but with additional intricate constraints. The Improved Discrete Bees Algorithm integrates swap and insert operators, detailed in Figure 3.

**Figure 4:**
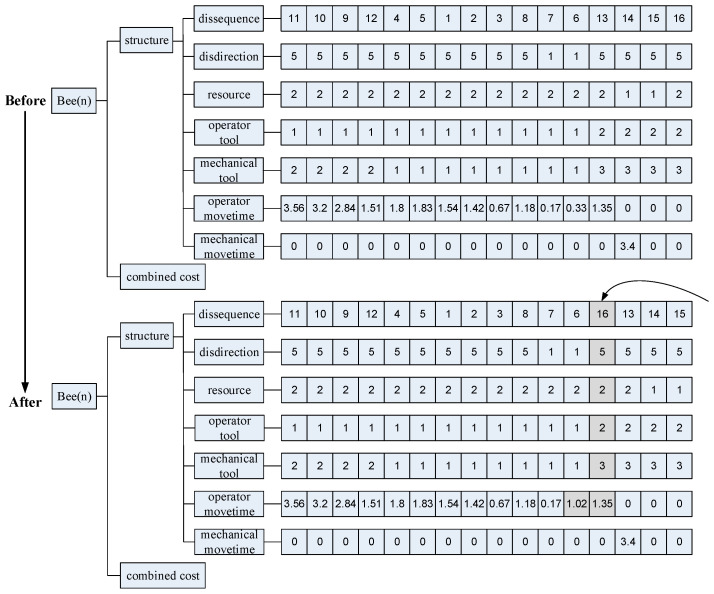
Insert operator in disassembly solution.

In combinatorial optimisation, the swap operator involves exchanging the positions of two elements or components within a solution to modify it. In disassembly sequencing, this means swapping the sequence of disassembled components. Adjustments are then made to the disassembly tool, direction, and movement time between these components. After applying the swap operator, it is essential to validate the resulting solution as a feasible disassembly plan. This verification involves a recursive check using an updated interference matrix. The insert operator details are provided in Figure 4.

In combinatorial optimisation, the insert operator entails choosing a position within a solution and inserting a new element, then rearranging other elements to accommodate this insertion. In disassembly sequence planning, the insert operator places a component’s disassembly order at a new position to create a fresh disassembly plan, necessitating adjustments to other parts of the plan. After generating a new plan, it is crucial to verify its feasibility as a viable disassembly solution.

#### 4.1.2. Genetic Crossover and Mutation

In IDBA, elite sites and best sites are identified similarly to EDBA. The distinction lies in the inclusion of a mutation operator in subsequent genetic operations. Once the elite sites and best sites are determined, all sites undergo pairwise crossover operations based on their combined costs. If a solution with a lower combined cost is found, it replaces the original solution. Following the completion of crossover operations, mutations are applied. Before executing mutations, a preferred direction is established to prioritise mutations in that direction. Initially, probabilities are set for both crossover and mutation operations in the genetic process.

Before initiating the crossover operation, it is necessary to have parental sequences. Then, a random encoding containing only 1 and 2 is generated. The codes are used to select the parts of the disassembly sequence that will be inherited by the offspring, as shown in Table 11. The crossover method employed in this article is according to Ref. [27]. In the context of disassembly sequence planning, the parental sequences refer to feasible disassembly plans. The sequences resulting from crossover are plans that contain features of the parental sequences. They may represent improved disassembly plans if those inherited features are the parents’ strong features.

**Figure 5:**
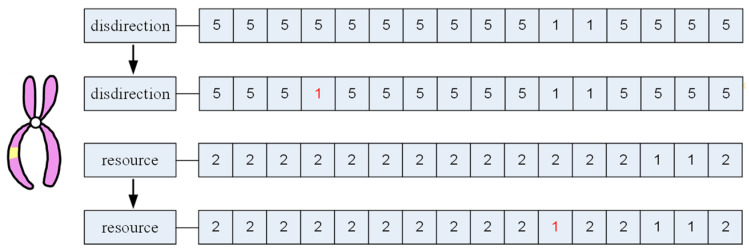
Genetic mutation in disassembly solution.

The mutation operator simulates genetic mutation observed in DNA and its implementation varies based on the problem’s specifics. In disassembly sequence planning, this operator adjusts disassembly directions and resources of components, influencing disassembly times. Figure 5 illustrates resource allocation and the specified preferred direction from Section 4.3. Following genetic crossover and mutation operations, the resulting plans must undergo validation for feasibility as disassembly solutions.

### 4.2. Disassembly Model

**Figure 6:**
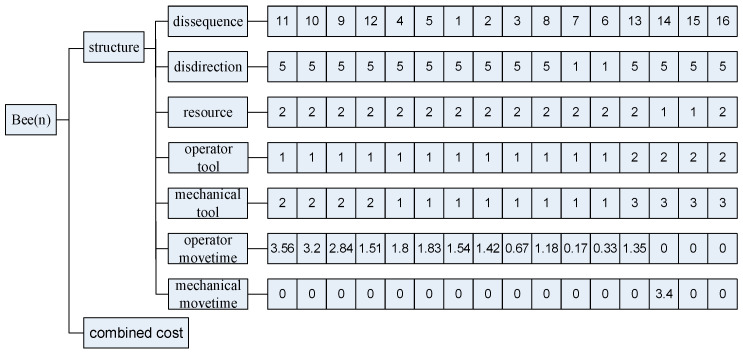
Structure and combined cost constituting individual bees.

A disassembly model has been developed using a feasible sequence generation method and an improved spatial interference matrix [15]. The improved interference matrix addresses the issue of infeasible solutions arising from bolted partial connections. The improved spatial interference matrix is derived based on the structural relationships between each component, resulting in six directional matrices (X+, X−, Y+, Y−, Z+, Z−). These matrices are utilised in conjunction with the feasible sequence generation method to obtain feasible disassembly solutions. Each generated individual bee represents a feasible disassembly solution. In this article, the bees are divided into two components: structure and combined cost, which is shown in Figure 6. The structure includes the disassembly sequence of components, the orientation of disassembled components, the disassembly resource of each component, the operator and robot disassembly tool of each component, and the movement time between adjacent components. For this work, the combined cost of a bee is the general value shown in Equation (19).

### 4.3. Forbidden Direction and Preferred Direction

If the disassembly direction of the generated feasible disassembly solutions is not constrained relative to the disassembly site and the conditions of the EoL products, the generated disassembly solutions may have some unfeasible disassembly directions. For example, the parts of an EoL product placed on the conveyor belt cannot be disassembled in the bottom direction of the product.

**Figure 7:**
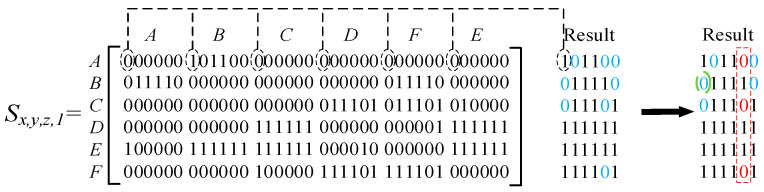
The setting of the forbidden direction.

Unreasonable disassembly directions will not occur in feasible disassembly sequence solutions when the forbidden direction is employed. The process involves merging the interference matrices from six directions into a comprehensive interference matrix. This resulting matrix, with dimensions (*n* × 6), where (*n*) represents the number of components in the disassembly target, categorises each column by direction (X+, X−, Y+, etc.). Elements within the matrix are either 0 or 1: a 0 indicates feasible disassembly in the specified direction. To generate feasible disassembly sequences, the approach randomly selects one zero-value element per iteration from this matrix, determining both the disassembly component and its corresponding direction for that iteration. When taking zero values from the result matrix, set a column where the zero value cannot be selected. This ensures that feasible disassembly sequences containing that direction are not generated, as shown in Figure 7. Zero values in result matrix are displayed in blue and the column where the zero values cannot be selected are displayed in red.

In complex products with multiple disassembly directions, different disassembly sequences can obtain optimal solutions depending on the preferred direction for disassembly operations. Setting a preferred direction helps achieve the optimal disassembly solution and aids in planning obstacle avoidance paths. This approach ensures efficient disassembly moves by selecting the most convenient direction of disassembly operations. The mutation operator is employed to mutate the direction in the feasible disassembly solution (individual bee) according to the preset mutation rate. In the direction mutation process, priority is given to mutating into the preset preferred direction (directions X+, X−, Y+, Y−, Z+, and Z− use the numbers 1, 2, 3, 4, 5, 6 instead).

### 4.4. Optimisation Objectives

#### 4.4.1. Time Cost

**Figure 8:**
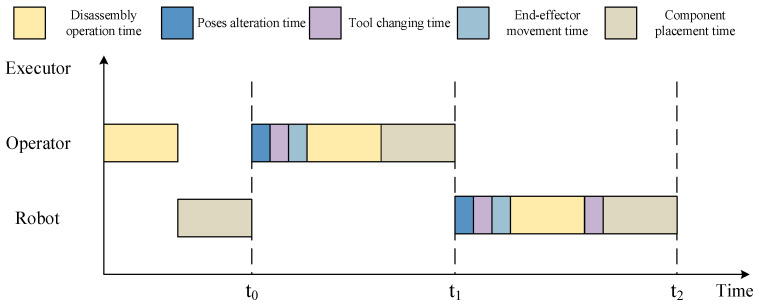
The Gantt chart of HRCD.

After obtaining a feasible disassembly sequence and assigning the disassembly tasks, it is necessary to calculate the total disassembly time required for this solution. After each component is disassembled, it should be placed immediately. The total disassembly time consists of the disassembly operation time, pose alteration time, tool changing time, end-effector movement time, and component placement time, as shown in Figure 8.

The total disassembly time of each solution can be evaluated by Equation (11).

(11) $$T=\sum_{i=1}^{n-1}bt(x_{i})+\sum_{i=1}^{n-1}pt(x_{i})+\sum_{i=1}^{n-1}dt(x_{j},x_{i+1})+\sum_{i=1}^{n-1}tt(x_{i},x_{i+1})+\sum_{i=1}^{n-1}mt(x_{i},x_{i+1})$$

bt represents the disassembly operation time of each component. The last component does not need to be disassembled, so the number of components that need to be disassembled is *n* − 1.

pt represents the component placement time. The placement tasks could be divided into two types, which are robot placement and operator placement. The time for robot placement and operator placement is uniformly set as 4 s and 3 s, respectively.

dt is the robot pose change time, as expressed in Equation (12). If the disassembly direction of the component is the same as that of the previous component, the time taken is 0. $t_{90}$ and $t_{180}$ are the pose alteration times when the disassembly direction changes to 90 degrees and 180 degrees, respectively.

(12) $$dt_{i}=\left\{\begin{array}{cc} 0 & dir\_c=0^{{}^{\circ}}\\ t_{90} & dir\_c=90^{{}^{\circ}}\\ t_{180} & dir\_c=180^{{}^{\circ}} \end{array}\right.$$

*TT* represents the time for switching disassembly tools. The tool changing time can be determined using the matrix in Equation (13). As the time required for tool disassembly and installation is different, the order of tool changing may affect the switching time due to the asymmetry of the tool change matrix. Moreover, the tool changing time for the operator and robot is also different. The article assumes that the tool changing time for operators is the same.

(13) $$TT=\begin{array}{c} T_{1}\\ T_{2}\\ \ldots \\ T_{n} \end{array}\overset{\begin{array}{cccc} T_{1} & T_{2} & \ldots & T_{n} \end{array}}{\left[\begin{array}{cccc} 0 & tt_{1,2} & \ldots & tt_{1,n}\\ tt_{2,1} & 0 & \ldots & tt_{2,n}\\ \ldots & \ldots & \ldots & \ldots \\ tt_{n,1} & tt_{n,2} & \ldots & tt_{n,n} \end{array}\right]}$$

$mt_{i,j}$ represents the movement time of an end effector between components. The end-effector movement time can be calculated using a distance matrix *MT*. Because there are two different disassembly resources, there are two sets of matrices corresponding to the operator and robot, respectively. The matrix is an *n*-dimensional square matrix representing the time taken for the end effector to move from one component to another. Because the movement time between components is independent of the order of adjacent components, this matrix is symmetric. The movement time in the matrix can be calculated by using the Euclidean distance, which is described by Equations (14) and (15). *v* represents the velocity of the end effector on a robot or the operator’s hand.

(14) mti,j=(xi−xj)2+(yi−yj)2+(zi−zj)2v

(15) $$MT=\begin{array}{c} C_{1}\\ C_{2}\\ \ldots \\ C_{n} \end{array}\overset{\begin{array}{cccc} C_{1} & C_{2} & \ldots & C_{n} \end{array}}{\left[\begin{array}{cccc} 0 & mt_{1,2} & \ldots & mt_{1,n}\\ mt_{1,2} & 0 & \ldots & mt_{2,n}\\ \ldots & \ldots & \ldots & \ldots \\ mt_{1,n} & mt_{2,n} & \ldots & mt_{n,n} \end{array}\right]}$$

#### 4.4.2. Payment Cost

The payment cost of human operators and robots consists of different parts. The payment of human operators mainly contains two parts: The first is the salary for human operators. Taking the data from the World Bank and the average salary from the automotive industry in North America, the payment for a disassembly operator is 45,620 dollars per year. Assuming the operators work 12 months per year, 22 days per month, and 8 h per day, the payment per second for a human operator (*P_salary_*) is 0.006 dollars per second. Another cost is from the payment for professional training due to the collaboration with robots requiring specific training, like safety training. Assuming the annual cost for training operators is 8000 dollars, the total cost for human operators in disassembly tasks is 0.0071 dollars per second. Equation (16) shows the definition of payment for human operators $P_{h}$, where $N_{h}$ represents the number of working hours per year.

(16) $$P_{h}=P_{salary}+\frac{C_{train}}{3600N_{h}}$$

For robots, the cost contains three different parts. The first is the payment for purchasing equipment. The price of a typical industry HRC robot ranges from 30,000 dollars to 80,000 dollars. In this report, assume the cost for the robot is 50,000 dollars, and assume the service life of robots is 3 years. The other cost is daily maintenance; assume it takes a worker three months to perform maintenance during the service period in an ideal situation, costing around 11,400 dollars. The final part is the electricity cost. For most HRC robots, the average power is no more than 1 kW. Assume the power of the robot is 1 kW, and the average electricity price in the UK is 20 *p*/kW, around 0.26 dollars per hour. The robot cost *P_r_* is defined in Equation (17), where $N_{y}$ represents the robot’s service time (year).

(17) $$P_{r}=P_{electricity}+\frac{C_{equipment}+C_{ma\mathrm{int}enance}}{3600N_{y}N_{h}}$$

The total payment in a disassembly solution is defined in Equation (18).

(18) $$P=\sum \left(P_{h}T_{i}^{h}+P_{r}T_{i}^{r}\right)$$

#### 4.4.3. General Evaluation Criterion

A general function is defined to evaluate the performance of the designed scheduling by calculating the trade-off between *T*, *P*, and *U*. By setting different coefficients, the emphasis of the disassembly solution can be changed. The combined cost (CC) is defined as Equation (19):

(19) $$\mathrm{CC}=w_{1}T+w_{2}P+w_{3}(100-U)$$

## 5. Case Studies

### 5.1. Case Study Selection

With the increasing popularity of new energy vehicles, the management of waste new energy vehicles has become an urgent issue that needs to be addressed by society. Remanufacturing is considered an excellent option for handling waste new energy vehicles, as many EoL products present in these vehicles can be processed through remanufacturing. The research on the remanufacturing of EoL components from waste new energy vehicles is of significant importance for resource conservation and environmental protection. Electric motors and EV power batteries are both important components of new energy vehicles, with electric motors categorised as smaller components and EV power batteries classified as larger components. In this section, case studies will be conducted on the task allocation and sequence planning of disassembly for these two components, which differ significantly in size.

### 5.2. Case Study 1: Electric Motor

**Figure 9:**
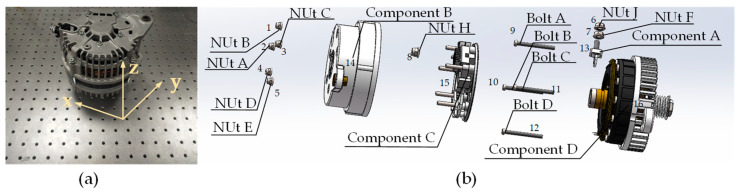
Photograph and exploded view of an electric motor. (**a**) Photograph. (**b**) Exploded view.

Section 5.2 will explore a case study on the task allocation and sequence planning of disassembly for electric motors, which will be treated as representative of smaller-sized EoL components in waste new energy vehicles. Photos of the electric motor is shown in Figure 9a, and its 3D exploded view is shown in Figure 9b. The disassembly information is listed in Table 12. Table 12 shows the disassembly points for each disassembly components, the corresponding disassembly times for the robot and human operator, and the disassembly tools for the robot and human operator. The component placement time for robots is 4 s, and for human operators, it is 3 s. In Equation (14), the value of v is set to 25 mm/s for the robot, and 30 mm/s for the human operator. Additionally, robots require grippers for placing components, whereas human operators do not use tools for placing components.

The tool changing time matrix can be expressed in Equation (20). The different sequence of replacement between the two tools also leads to differences in replacement time, so this matrix is not a symmetric matrix. Human operator tool changing time is 4 s.

(20) $$TT=\begin{array}{c} Nu\\ Sc\\ Gr \end{array}\overset{\begin{array}{ccc} Nu & Sc & Gr \end{array}}{\left[\begin{array}{ccc} 0 & 6 & 7\\ 5 & 0 & 8\\ 9 & 10 & 0 \end{array}\right]}$$

Assuming that the pose alter time is 0, 1 s, or 2 s, the matrix of the robot pose alteration time can be expressed in Equation (21).

(21) $$dt_{i}=\left\{\begin{array}{cc} 0 & dir\_c=0^{{}^{\circ}}\\ 1 & dir\_c=90^{{}^{\circ}}\\ 2 & dir\_c=180^{{}^{\circ}} \end{array}\right.$$

**Figure 10:**
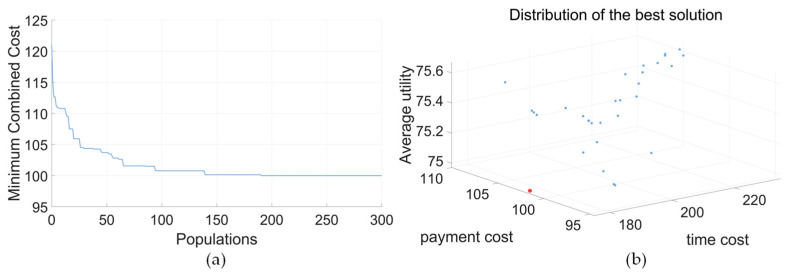
Iterative diagram (electric motor, balance mode). (**a**) Minimum combined cost. (**b**) Iterative scatter plot.

Due to the prohibited and preferred directions reflected in the disassembly directions, the optimal solution is different. The iterative results of the minimum combined cost for IDBA are shown in Figure 10. In Figure 10a, the lines represent iterative changes. In Figure 10b, the points denote different solutions of the iterations, with the red point indicating the optimal solution. As shown in Table 13, for the electric motor, the setting of the prohibited and preferred directions directly affects the disassembly order of the parts, and the disassembly time is increased for the optimal solution.

**Figure 11:**
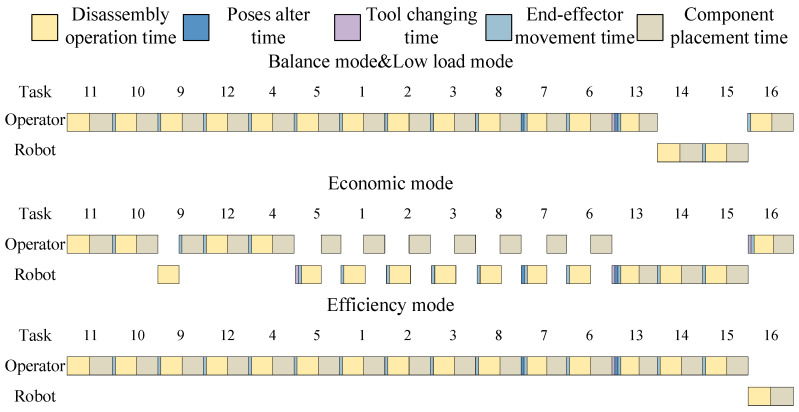
The Gantt chart of the optimal disassembly solution of the electric motor.

The results of different modes are all displayed in Table 14 and Table 15. For all modes of the electric motor, the prohibited direction is Z−, and the preferred direction is Z+. The Gantt chart of the optimal solution of the electric motor is shown in Figure 11.

As shown in Figure 11, the optimal disassembly solution is the same for both the balance mode and low-load mode. The disassembly solutions differ significantly across different modes, indicating that varying emphases in multi-objective optimisation lead to different optimisation results.

### 5.3. Case Study 2: EV Power Battery

**Figure 12:**
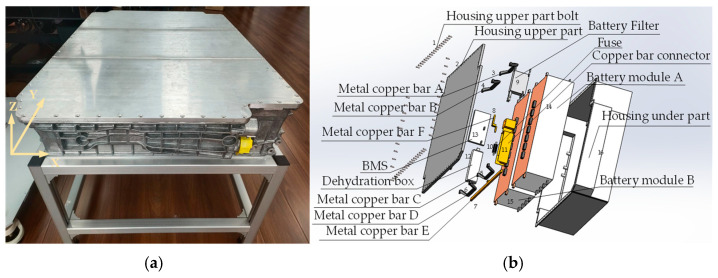
Photograph and exploded view of the power battery. (**a**) Photograph. (**b**) Exploded view.

In Section 5.3, a case study on the task allocation and sequence planning of disassembly for EV power batteries will be conducted, and this study will consider EV power batteries as representatives of the larger-sized EoL components in waste new energy vehicles. Photos of the power battery is shown in Figure 12a, and its 3D exploded view is shown in Figure 12b. The disassembly information is listed in Table 16.

**Figure 13:**
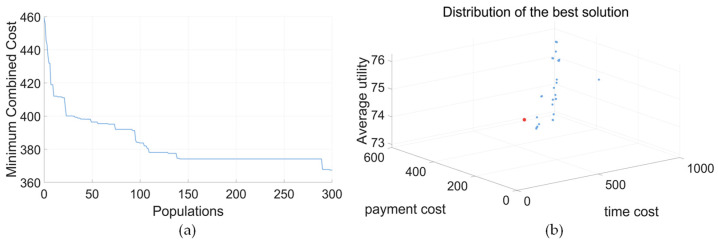
Iterative diagram (power battery, balance mode). (**a**) Minimum combined cost. (**b**) Iterative scatter plot.

The iterative results of the minimum combined cost for IDBA are shown in Figure 13. In Figure 13a, the lines represent iterative changes. In Figure 13b, the points denote different solutions of the iterations with the red point indicating the optimal solution. For the power battery, due to its structural uniqueness, setting the prohibited and preferred directions directly did not affect the disassembly sequence, direction, and resource of the components, which is shown in Table 17. Therefore, the prohibited and preferred directions are additional constraints in the disassembly sequence planning problem.

**Figure 14:**
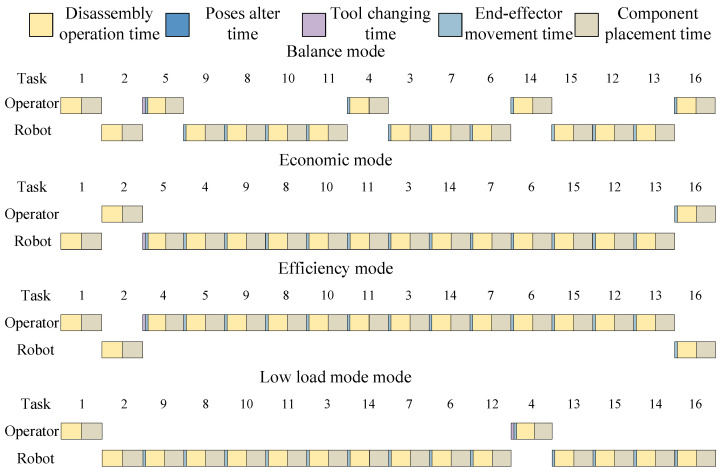
Gantt chart of the optimised disassembly solution of the power battery.

The results of different modes are all displayed in Table 18 and Table 19. For all modes of the power battery, the prohibited direction is Z−, and the preferred direction is Z+. The Gantt chart of the optimal solution of a power battery is shown in Figure 14.

### 5.4. Performance Analysis

The genetic part of IDBA was added based on EDBA, incorporating the existing genetic operators and introducing a crossover operator in the genetic part. EDBA was programmed according to Ref. [15]. The IDBA parameters are shown in Table 20.

**Figure 15:**
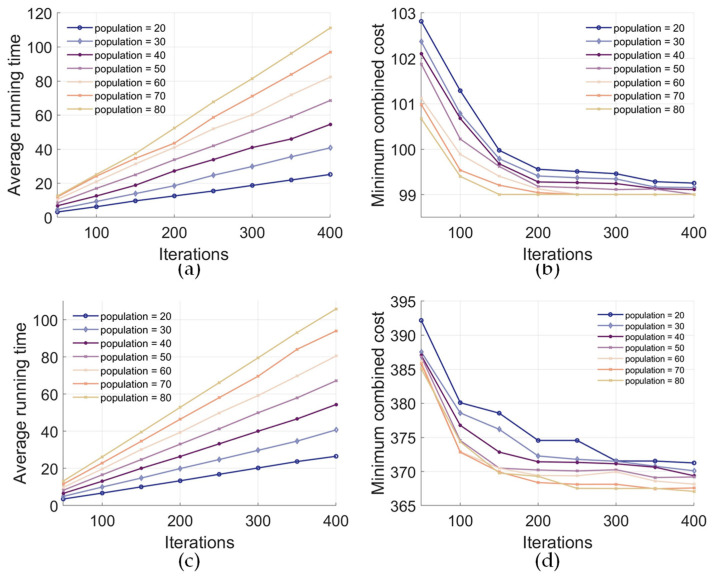
IDBA performance for different population sizes and iterations. (**a**) Average running time (electric motor). (**b**) Minimum combined cost (electric motor). (**c**) Average running time (power battery). (**d**) Minimum combined cost (power battery).

Each data set had an average of fifty experimental runs; the mode type was balance mode. The maximum number of iterations varied from 50 to 400, and the population size ranged between 20 and 80. In Figure 15a,c, the average running time of the program gradually increases with increasing iterations and population. Figure 15b,d show that the minimum combined cost decreases with increasing iterations and population. A larger population reduces the minimum combined cost when the iteration count is fixed. This is because a larger population increases the likelihood of discovering superior solutions, facilitated by deploying more scouting bees after site selection.

**Figure 16:**
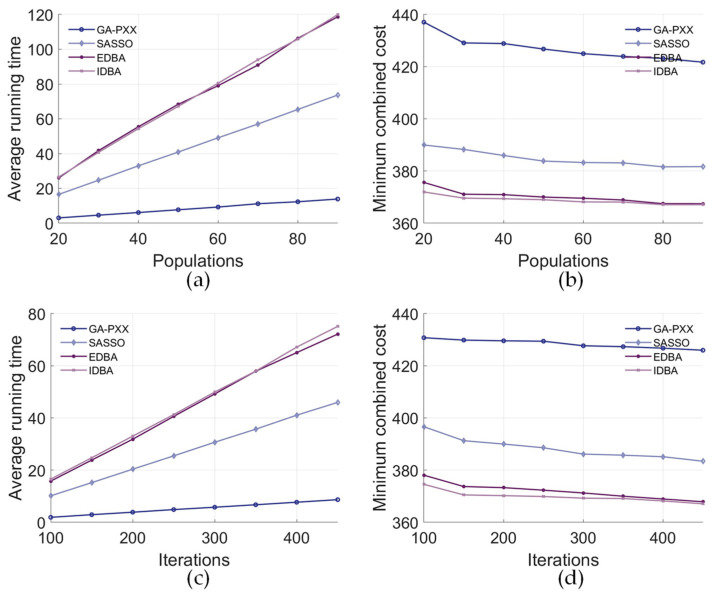
Performance comparisons of different optimisation algorithms (power battery case study). (**a**) Average running time for different population sizes. (**b**) Minimum combined cost for different population sizes. (**c**) Average running time for different numbers of iterations. (**d**) Minimum combined cost for different numbers of iterations.

Figure 16 presents performance comparisons between the proposed algorithm for disassembly sequence planning and other optimisation algorithms. Each data point represents the average result from fifty experiments. The maximum number of iterations ranged from 100 to 450, and the population size varied between 20 and 90. The comparison included IDBA, EDBA, a genetic algorithm with a priority-based protection crossover operator (GA-PPX) [27], and a self-adaptive simplified swarm optimisation algorithm (SASSO) [30]. A consistent forbidden direction was set for generating bees in each algorithm. For EDBA and IDBA, the selection site was set to half of the number of scout bees, the elite site was set to one-tenth of the number of scout bees, the number of employed bees for the elite site was set to ten, and the best site was set to five. The crossover probability for GA-PPX was set to 0.8. The parameters of SASSO automatically changed according to the conditions of the individuals. For the power battery, the GA had a shorter average running time, as shown in Figure 16a,c. GA-PPX and the SASSO had longer minimum combined costs, as shown in Figure 16b,d. Compared to EDBA, IDBA yielded a shorter minimum combined cost and better performance in obtaining the optimal disassembly solution.

## 6. Conclusions

HRCD has been intensively researched to improve efficiency and reduce costs. In this paper, a method of task allocation and sequence planning for human–robot collaborative disassembly using the Bees Algorithm was investigated. Case studies involving an electric motor and a power battery were carried out to validate the proposed method. The main contributions of our work are as follows:

(1) Having different classification criteria for operators and robots makes task allocation more reasonable. The criteria proposed in the article facilitate the allocation of disassembly tasks to more suitable disassembly resources, thereby achieving optimal disassembly solutions.
(2) Integrating both forbidden directions and preferred directions that align with practical needs into the disassembly sequence planning makes the disassembly planning more realistic. The proposed method can generate optimal solutions for HRCD because practical application scenarios are considered by setting forbidden and preferred directions.
(3) The article proposes the IDBA algorithm. In comparison with EDBA, GA-PPX, and SASSO, IDBA has better performance in obtaining an optimal disassembly sequence to improve disassembly efficiency. Compared with EDBA, performing additional genetic crossover and mutation operations on elite sites increases the probability of finding the optimal disassembly solution.
(4) During disassembly sequence planning, the placement of disassembled components was considered, aligning the disassembly process with reality.

The limitations of this work include the following: (1) Keeping the placement time of disassembled components constant might result in differences between the overall calculated disassembly time and the actual disassembly time because the placement times of different components vary with the weight of the components and the different placement locations. (2) The power battery was simplified, and the wiring harnesses and fasteners were ignored. This method does not apply to highly complex components such as wire harnesses. (3) In serial disassembly with collaborative robots, the time interval between disassembly and placement in the proposed method was not considered. (4) For an EoL product with a large size, the reach of the robot’s and operator’s arms should be considered. (5) The proposed method involves the disassembly resources of an operator and a robot. For complex products with more disassembly resources, the evaluation criteria and optimisation will be complex. In the future, more disassembly resources will be considered, and specific task allocation strategies for different situations, for example, one involving two robots and one operator, will be developed. Parallel and partial disassembly sequence planning will also be investigated.

Although the method demonstrated excellent performances in the current setting, its potential for scalability and broad applicability warrants further investigation. The advantage of the method lies in its efficient algorithm design and flexible parameter adjustments, which endows it with a certain degree of generalisability when optimising different types of EoL products. Future research could explore the application of this method to various types of EoL products to validate its effectiveness in more scenarios. Additionally, due to the computational resource requirements, the algorithm could be optimised to reduce computational complexity.

## Figures and Tables

**Table 1:** Preliminary assessment for subtasks allocated to human operators [37].

Safety and Capability Assessment	Assessment Description
Toxic material or components	The material of disassembly components is toxic, or toxic substances will be produced during the disassembly process.
Unsuitable operation environment	The disassembly operation environment is not suitable for human operators, due to conditions like temperature.
Exceed human limit	The weight or the size of the components exceeds human operators’ limit.
Exceed human disassembly capability	The disassembly task cannot be handled by human operators due to their capability, even with specific tools.
Unsafe robots	The robots that work with human operators do not meet the latest safety standards.

**Table 2:** Preliminary assessment for subtasks allocated to human operators [38].

Item	Unit	Description
MVC	N	Maximum capacity of muscle
$F_{cem}(t)$	N	Current exerted maximum force, the current capacity of muscle
$F_{load}(t)$	N	An external load of muscle, the force that the muscle needs to generate
k	$\min^{-1}$	Constant value, 1
U	min	Fatigue index
$f_{MVC}$	N/A	The ratio of $F_{load}$ to MVC
MET	min	The duration for $F_{cem}$ to decrease to the current $F_{load}$
MAT	min	The maximum endurance time in a specific load

**Table 3:** Basic fitness value standards [40].

Posture Type	Description		Fitness Value: Duration of Evaluation Period for Static Movement of Trunk/Arms in % of Time				
Time Duration in Each Posture/Total Time (%)			2 to 10	11 to 20	21 to 33	34 to 67	68 to 100
Standing		Upright slightly bent forward	1	1	1	0.95	0.9
		Bend forward	0.95	0.9	0.85	0.7	0.6
		Bend deeply forward	0.95	0.85	0.8	0.6	0.4
		Upright arms at/above shoulder level	0.92	0.8	0.7	0.55	0..3
		Upright arms at/above head level	0.9	0.8	0.65	0.3	0
Sitting		Upright slightly bent forward	1	1	1	0.95	0.9
		Bend forward	0.98	0.94	0.9	0.85	0.6
		Upright arms at/above shoulder level	0.95	0.88	0.8	0.65	0.4
Kneeling		Upright arms at/above head level	0.92	0.84	0.7	0.55	0.25
		Upright slightly bent forward	0.95	0.87	0.8	0.65	0.45
		Bend forward	0.91	0.83	0.72	0.52	0.25
		Arms at/above shoulder level	0.87	0.75	0.6	0.2	0
Lying		Lying	0.84	0.79	0.55	0.3	0

**Table 4:** Fitness value standards of twist factor, lateral factor, and reach factor in ergonomics [40].

Fitness Values of the Twist Factor, Lateral Factor, and Reach Factor						
Twist level fitness value (${\mathrm{degree}}^{{}^{\circ}}$)			Twist time fitness value (percentage)			
0 to 30	30 to 60	60 to 90	1 to 6	6 to 15	15 to 30	30 to 100
1	0.6	0.2	0.8	0.6	0.5	0.4
Lateral level fitness value (${\mathrm{degree}}^{{}^{\circ}}$)			Lateral time fitness value (percentage)			
0 to 15	15 to 30	Over 30	1 to 6	6 to 15	15 to 30	30 to 100
1	0.7	0.3	0.8	0.6	0.5	0.4
Reach level fitness value (${\mathrm{degree}}^{{}^{\circ}}$)			Reach time fitness value (percentage)			
0 to 60	60 to 80	80 to 100	1 to 6	6 to 15	15 to 30	30 to 100
0.9	0.8	0.5	0.8	0.6	0.5	0.4
Total fitness value of twist/lateral/reach factor = 0.5 × (level fitness value + time fitness value)						

**Table 5:** Standards to calculate the fitness value of material handling in a disassembly task [40].

Material Handling Numeric Evaluation				
Design Attribute	Design Feature	Design Parameters	Interpretation	Fitness Value
Material handling	Component size	Component dimensions	The component is easy to grasp or hold	0.8
			The component is moderately difficult to grasp or hold	0.5
			The component is difficult to grasp	0.3
		Magnitude of weight	Light component (<3.5 kg)	0.8
			Moderately heavy component (<8 kg)	0.5
			Heavy component	0.3
	Component symmetry	Symmetry and operational difficulty of components	Light and symmetric component	0.9
			Light and semi-symmetric component	0.85
			Light and asymmetric component	0.75
			Moderately heavy and symmetric component	0.7
			Moderately heavy and semi-symmetric component	0.6
			Moderately heavy and asymmetric component	0.55
			Heavy and symmetric component	0.3
			Heavy and semi-symmetric component	0.2
			Heavy and asymmetric component	0

**Table 6:** Evaluation criteria to calculate the fitness value of difficulty and flexibility in a disassembly task [41].

Difficulty and Flexibility Assessment				
Design Attribute	Design Feature	Design Parameters	Interpretation	Fitness Value
Requirement of tools for disassembly	Exertion of force Exertion of torque	The complexity of using tools and the number of tools used	No tools required	1
			Single common tool required	0.8
			Multiple common tools required	0.6
			Single special tool required	0.5
			Multiple special tools required	0.3
Accessibility of joints/grooves	Dimensions	Length, breadth, depth, radius, angle relative to the surface	Shallow and broad fastener recesses, large and readily visible slot/recess in case of snap fits	1
			Deep and narrow fastener recesses, obscure slot/recess in case of snap fits	0.6
			Very deep and very narrow fastener recesses, slot for prying open snap fits difficult to locate	0.4
	Location	On plane surface	Groove location allows easy access	1
		On angular surface	Groove location is difficult to access, some manipulation required	0.6
		In a slot	Groove location is very difficult to access	0.4
Positioning	Level of accuracy required to position the tool	Symmetry	No accuracy required	1
			Limited accuracy required (less than 0.1 mm)	0.7
			High accuracy required (less than 0.01 mm)	0.1
		Asymmetry	No accuracy required	0.8
			Limited accuracy required (less than 0.1 mm)	0.5
			High accuracy required (less than 0.01 mm)	0

**Table 7:** Standards to calculate the fitness value of repetition in a disassembly task.

Repetition Evaluation				
Design Attribute	Design Feature	Design Parameters	Interpretation	Fitness Value
Repetition	The number of repetitions on the same task	Repetitions within a single task	Less than 10 times	0.8
			From 10 times to 20 times	0.6
			From 20 times to 50 times	0.3
			More than 50 times	0
		Repetitions across multiple tasks	Less than 10 times	0.8
			From 10 times to 20 times	0.5
			From 20 times to 50 times	0.1
			More than 50 times	0

**Table 8:** Common tools and corresponding disassembly operation.

Tool and Corresponding Capability of Robot Assessment								
Capacity /Tool	Blocking	Grasping	Removing	Rotating	Deforming	Unscrewing	Pulling/ Pushing	Dismantling
Chucks	√							
Grippers		√	√					
Pliers			√	√	√			
Spanners				√		√		
Extractor							√	
Punches							√	
Hammers							√	
Drills								√
Nut runner						√		

**Table 9:** Difficulty assessment in disassembly task and corresponding fitness value.

Task Interpretation	Fitness Value
Disassembly task requires the end effector of the robot’s arm to move in one direction	1
Disassembly task requires the end effector of the robot’s arm to move in two directions	0.8
Disassembly task requires the end effector of the robot’s arm to move in more than two directions	0.6

**Table 10:** Corresponding fitness value.

Actual Distance/Robot’s Reachability (Percentage)	Fitness Value
0.1	0.15
0.2	0.34
0.3	0.49
0.4	0.64
0.5	0.9
0.6	1
0.7	0.95
0.8	0.68
0.9	0.38
1	0.1

**Table 11:** Example of crossover.

Parent 1	Parent 2	Mask of Child	Child
7	7	2	7
8	8	1	8
2	9	2	9
4	10	2	10
5	1	2	1
9	16	1	2
11	6	2	16
14	15	2	6
10	2	1	4
12	4	2	15
13	5	2	5
3	11	1	11
1	14	2	14
16	12	1	12
6	13	1	13
15	3	2	3

**Table 12:** Disassembly information for the electric motor.

EoL Products	Part	Disassembly Components	Disassembly Point (mm)	Robot bt/s	Robot Tool	Human bt/s	Human Tool
Electric motor	1	Nut A	(−36, 0, 153)	5	Nutrunner	4	Spanner
	2	Nut B	(−10, −38, 153)	5	Nutrunner	4	Spanner
	3	Nut C	(30, −23, 153)	5	Nutrunner	4	Spanner
	4	Nut D	(37, 35, 160)	5	Nutrunner	4	Spanner
	5	Nut E	(−15, 50, 160)	5	Nutrunner	4	Spanner
	6	Nut F	(80, −20, 145)	5	Nutrunner	4	Spanner
	7	Nut G	(85, −20, 145)	5	Nutrunner	4	Spanner
	8	Nut H	(50, −23, 150)	5	Nutrunner	4	Spanner
	9	Bolt A	(−12, 50, 130)	6	Screwdriver	5	Spanner
	10	Bolt B	(−68, −28, 130)	6	Screwdriver	5	Spanner
	11	Bolt C	(28, −75, 130)	6	Screwdriver	5	Spanner
	12	Bolt D	(70, 27, 130)	6	Screwdriver	5	Spanner
	13	Component A	(90, −20, 145)	10	Gripper	8	Gripper
	14	Component B	(60, 60, 160)	11	Gripper	9	Gripper
	15	Component C	(0, 0, 166)	16	Gripper	14	Gripper
	16	Component D	(50, −23, 150)	14	Gripper	12	Gripper

**Table 13:** Optimisation results (Balance mode).

Product	Setting	Attribute	Result
Electric motor	Forbidden direction (Z−) and preferred direction (Z+)	Sequence	11-10-9-12-4-5-1-2-3-8-7-6-13-14-15-16
		Direction	5-5-5-5-5-5-5-5-5-5-1-1-5-5-5-5
		Resource	2-2-2-2-2-2-2-2-2-2-2-2-2-1-1-2
	Without setting forbidden and preferred direction	Sequence	9-5-1-10-2-11-3-8-4-12-7-6-13-14-16-15
		Direction	5-5-5-5-5-5-5-5-5-5-1-1-5-5-6-6
		Resource	2-2-3-2-2-3-3-3-3-3-3-3-1-1-1-2

**Table 14:** The optimal results data of electric motor in four different modes.

Mode Type	Weight Attribute		Optimal Result Data of Electric Motor	
Balance mode	Time weight	0.33	Time (s)	176.805
	Payment weight	0.33	Payment (cent)	102.138
	Utility weight	0.33	Average utility	75.512
Economic mode	Time weight	0.15	Time (s)	200.385
	Payment weight	0.7	Payment (cent)	87.386
	Utility weight	0.15	Average utility	75.512
Efficiency mode	Time weight	0.7	Time (s)	171.782
	Payment weight	0.15	Payment (cent)	115.085
	Utility weight	0.15	Average utility	76.093
Low-load mode	Time weight	0.15	Time (s)	176.805
	Payment weight	0.15	Payment (cent)	102.138
	Utility weight	0.7	Average utility	75.512

**Table 15:** The results of the optimal solution in four different modes.

Mode	Product	Attribute	Result
Balance mode	Electric motor	Sequence	11-10-9-12-4-5-1-2-3-8-7-6-13-14-15-16
		Direction	5-5-5-5-5-5-5-5-5-5-1-1-5-5-5-5
		Resource	2-2-2-2-2-2-2-2-2-2-2-2-2-1-1-2
Economic mode	Electric motor	Sequence	11-10-9-12-4-5-1-2-3-8-7-6-13-14-15-16
		Direction	5-5-5-5-5-5-5-5-5-5-1-1-5-5-5-5
		Resource	2-2-3-2-2-3-3-3-3-3-3-3-1-1-1-2
Efficiency mode	Electric motor	Sequence	11-10-9-12-4-5-1-2-3-8-7-6-13-14-15-16
		Direction	5-5-5-5-5-5-5-5-5-5-1-1-5-5-5-5
		Resource	2-2-2-2-2-2-2-2-2-2-2-2-2-2-2-1
Low-load mode	Electric motor	Sequence	11-10-9-12-4-5-1-2-3-8-7-6-13-14-15-16
		Direction	5-5-5-5-5-5-5-5-5-5-1-1-5-5-5-5
		Resource	2-2-2-2-2-2-2-2-2-2-2-2-2-1-1-2

**Table 16:** Disassembly information for the power battery.

EoL Products	Part	Disassembly Components	Disassembly Point (mm)	Robot bt/s	Robot Tool	Human bt/s	Human Tool
Power battery	1	Housing upper part bolt	(522, 372, 224)	506	Screwdriver	362	Spanner
	2	Housing upper part	(522.5, 0, 224)	14	Gripper	12	Gripper
	3	Metal copper bar A	(40, 310, 204)	10	Gripper	8	Gripper
	4	Metal copper bar B	(685, 310, 204)	10	Gripper	8	Gripper
	5	Metal copper bar C	(685, 310, 204)	10	Gripper	8	Gripper
	6	Metal copper bar D	(40, 750, 204)	10	Gripper	8	Gripper
	7	Metal copper bar E	(40, 526, 124)	12	Gripper	10	Gripper
	8	Metal copper bar F	(320, 80, 199)	8	Gripper	6	Gripper
	9	Battery Filter	(470, 35, 199)	16	Gripper	14	Gripper
	10	Fuse	(240, 70, 134)	11	Gripper	9	Gripper
	11	Copper bar connector	(215, 40, 180)	15	Gripper	13	Gripper
	12	Dehydration box	(230, 1000, 199)	8	Gripper	6	Gripper
	13	Battery management system	(530, 1010, 206)	12	Gripper	10	Gripper
	14	Battery module A	(350, 310, 184)	17	Gripper	15	Gripper
	15	Battery module B	(350, 750, 184)	17	Gripper	15	Gripper
	16	Housing under part	(522.5, 0, 210)	14	Gripper-	12	Gripper

**Table 17:** Optimisation results (balance mode).

Product	Setting	Attribute	Result
Power battery	Forbidden direction (Z−) and preferred direction (Z+)	Sequence	1-2-5-9-8-10-11-4-3-7-6-14-15-12-13-16
		Direction	5-5-5-5-5-5-5-5-5-5-5-5-5-5-5-5
		Resource	2-1-2-1-1-1-1-2-1-1-1-2-1-1-1-2
	Without setting forbidden and preferred direction	Sequence	1-2-5-9-8-10-11-4-3-7-6-14-15-12-13-16
		Direction	5-5-5-5-5-5-5-5-5-5-5-5-5-5-5-5
		Resource	2-1-2-1-1-1-1-2-1-1-1-2-1-1-1-2

**Table 18:** The optimal results data of power battery in four different modes.

Mode Type	Weight Attribute		Optimal Result Data of Battery	
Balance mode	Time weight	0.33	Time (s)	706.598
	Payment weight	0.33	Payment (cent)	380.118
	Utility weight	0.33	Average utility	74.3449
Economic mode	Time weight	0.15	Time (s)	864.954
	Payment weight	0.7	Payment (cent)	248.837
	Utility weight	0.15	Average utility	75.694
Efficiency mode	Time weight	0.7	Time (s)	665.132
	Payment weight	0.15	Payment (cent)	459.103
	Utility weight	0.15	Average utility	73.9313
Low-load mode	Time weight	0.15	Time (s)	720.09
	Payment weight	0.15	Payment(cent)	369.257
	Utility weight	0.7	Average utility	75.188

**Table 19:** The results of the optimal solution in four different modes.

Mode	Product	Attribute	Result
Balance mode	Power battery	Sequence	1-2-5-9-8-10-11-4-3-7-6-14-15-12-13-16
		Direction	5-5-5-5-5-5-5-5-5-5-5-5-5-5-5-5
		Resource	2-1-2-1-1-1-1-2-1-1-1-2-1-1-1-2
Economic mode	Power battery	Sequence	1-2-5-4-9-8-10-11-3-14-7-6-15-12-13-16
		Direction	5-5-5-5-5-5-5-5-5-5-5-5-5-5-5-5
		Resource	1-2-1-1-1-1-1-1-1-1-1-1-1-1-1-2
Efficiency mode	Power battery	Sequence	1-2-4-5-9-8-10-11-3-14-7-6-15-12-13-16
		Direction	5-5-5-5-5-5-5-5-5-5-5-5-5-5-5-5
		Resource	2-1-2-2-2-2-2-2-2-2-2-2-2-2-2-1
Low-load mode	Power battery	Sequence	1-2-9-8-10-11-3-5-7-6-12-4-13-15-14-16
		Direction	5-5-5-5-5-5-5-5-5-5-5-5-5-5-5-5
		Resource	2-1-1-1-1-1-1-2-1-1-1-2-1-1-1-1

**Table 20:** Parameter design.

Parameters	Value
Iteration	50–400
Scout bee	20–80
Selected site	Scout bee/2
Elite site	Scout bee/10
Elite site bee	10
Selected site bee	5
Mutation rate	0.8
Crossover rate	0.8

## Data Availability

The raw data supporting the conclusions of this article will be made available by the authors on request.
